# Correlates of institutionalized senior veterans' quality of life in Taiwan

**DOI:** 10.1186/1477-7525-8-70

**Published:** 2010-07-17

**Authors:** Hsiao-Ting Chang, Li-Fan Liu, Chun-Ku Chen, Shinn-Jang Hwang, Liang-Kung Chen, Feng-Hwa Lu

**Affiliations:** 1 Institute of Gerontology, College of Medicine, National Cheng Kung University, Tainan, Taiwan; 2 Department of Family Medicine and Center for Geriatrics and Gerontology, Taipei Veterans General Hospital, and National Yang-Ming University School of Medicine, Taipei, Taiwan; 3 Department of Radiology, Taipei Veterans General Hospital, and National Yang-Ming University School of Medicine, Taipei, Taiwan; 4 Department of Family Medicine, College of Medicine, National Cheng Kung University, Tainan, Taiwan

## Abstract

**Background:**

Senior veterans living in government sponsored, long-term care (LTC) facilities, known as veterans' homes (VHs), are a special minority group in Taiwan. These seniors came from different provinces of mainland China during their teenage years at the end of civil wars in 1945. The situation of institutionalized senior veterans shares many characteristics with the concept of "total institution". Very little quality of life (QOL) research has involved senior veterans. This study aimed to explore the QOL and related factors of VH-dwelling senior veterans in Taiwan.

**Methods:**

Chronic conditions and socio-demographic characteristics of 260 male VH residents were recorded. The Brief Form of the World Health Organization's Quality of Life Questionnaire (WHOQOL-BREF, Taiwanese version); Short-Form 36; Inventory of Socially Supportive Behavior questionnaire; Geriatric Depression Scale-short form; Barthel Index; and instrumental activities of daily living were used. Data analyses including descriptive and inferred statistics were performed using SPSS, version 17.

****Results**:**

WHOQOL-BREF showed acceptable reliability in this study. Compared to Taiwanese male norms, WHOQOL-BREF physical, psychological, and social relationship domain scores were around the 25th percentile, and the environment domain was about the 75th percentile. Our participants scored low in all concepts of SF-36. Although these residents rated the social support of their children, relatives, friends, social and medical staff as low, they gave high satisfaction ratings to their social supports. On multiple stepwise linear regression analysis, depressive symptoms, number of chronic conditions, retired military rank, and relatives' support correlated with QOL in both the physical and psychological domains. Friends' support and depressive symptoms correlated with the social relationships domain. Friends' support and instrumental activities of daily living correlated with the environment domain.

**Conclusions:**

In general, institutionalized senior veterans' QOL was lower than Taiwanese male norms. Helping senior veterans to effectively improve their subjective mental health and social support, and controlling chronic disease appears to be critical to their QOL.

## Background

Taiwan is an economically well-developed island located in the Asia-Pacific region with a population exceeding 2.3 million. As of December 2008, 10.4% of the inhabitants were elderly [1]. A special group of elderly, known as "diasporas veterans", originating from different provinces of mainland China, account for around 12% of this aged population. Some live in government-sponsored, long-term care (LTC) facilities - known as veterans' homes (VHs) [2]. The VHs were built for the care of veterans who were injured in World War II or the following Civil War between the Kuomintang and the Chinese Communist Party, or those who were unable to work. VHs in Taiwan share many characteristics with those of "total institution"; a concept framed by Goffman [3], which has several characteristics, including disappearance of private life, life in common, planned and supervised activities, inmate/staff division, and self-mortification. In other words, residents are isolated from the outside world and live with a different set of rules and norms.

In these VHs, personal care or nursing services are provided according to senior veterans' activities of daily living (ADL), as assessed by their Barthel Index scores [4]. Residents who are capable of their own personal care live in the independent domiciliary units. However, if a resident has a Barthel Index lower than 90, or needs skilled nursing care, they will be assigned to the disabled nursing care units. All veterans living in a VH can receive financial support for monthly expenses from the government, amounting to around 417 USD per month. Basically, lodging is free; however, residents need to pay for their own meals (about 100 USD per month) [4].

Quality of life (QOL) is one of the central issues in caring for the elderly [5,6], but very little attention has been paid to those who are institutionalized [7-9], especially these senior veterans living in VHs in Taiwan [9]. There are several factors that are believed to influence the QOL of community-dwelling elderly [7,10,11]. A study to explore the QOL and health utility of 465 residents in LTC institutions showed that the World Health Organization Quality of Life-BREF (WHOQOL-BREF) is useful for evaluating health-related QOL of conscious institutionalized elderly [7]. A recent article used the Euroqol 5 D questionnaire to check the relationship of QOL to dispositional optimism, health locus control, and self-efficacy between community-dwelling, veterans' housing and a LTC home elderly in Poland. The study reported that veterans' home elderly should be the primary focus population for improving QOL [10]. However, to the best of our knowledge, there has been no comprehensive evaluation of institutionalized elderly veterans in Taiwan. Thus, the aim of this study was to evaluate subjective and objective physical and mental health, functional status and social health, and examine the QOL of these institutionalized elderly veterans receiving personal care in southern Taiwan, and to identify the correlates of their QOL.

## Methods

### Ethical statement

This study was approved by the Human Experiment and Ethics Committee of National Cheng Kung University Hospital, and subjects were interviewed after their informed consent was obtained.

### Settings and participants

**Figure 1:**
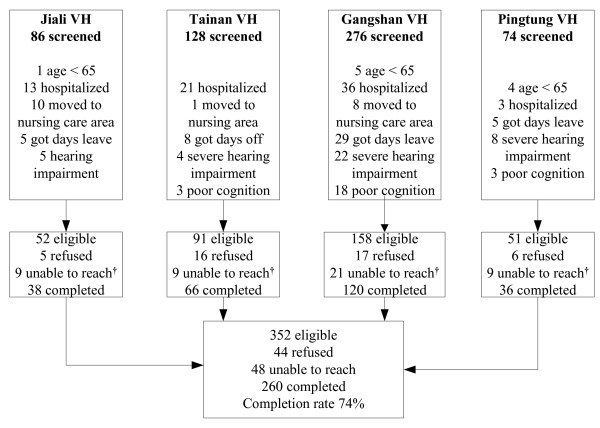
**Participants flow chart**. * Poor cognition: the MMSE score lower than 15 for education level of illiteracy or lower than 24 for literacy. **^† ^**Unable to reach: veterans did not meet during the survey period.

At the end of 2008, a total of 4,751 veterans were receiving personal care in 14 VHs in Taiwan [11]. Male veterans are randomly assigned to VHs, while the very few female veterans are assigned to a separate building in one VH in Tainan. From a medical perspective, all VHs in Taiwan are attributed to the administrative divisions of three tertiary veterans' general hospitals, located in northern, middle or southern Taiwan and regulated under the Veterans Affairs Commission. We selected four VHs belonging to one of the tertiary veterans' general hospital; Kaohsiung Veterans' General Hospital, located in southern Taiwan. All the buildings in the four VHs were constructed with a similar symmetrical architecture. On each floor, there is a central nursing station surrounded by residents' rooms. We applied a cluster sampling method to select one of the two independent domiciliary units on each floor thus composed of 50% veterans in every VH. Overall, there were 352 eligible male subjects, of which 260 residents ≥ 65 years of age were recruited. Those with poor cognitive function, a Mini-Mental Status Examination (MMSE) score lower than 15 for an education level of illiterate or lower than 24 for literate [12,13], severe hearing impairment, or those who had been admitted to the VH for less than 3 months were excluded. A diagram of the study population is shown in Figure 1.

### Demographic data

Participants' age, gender, birthday, education level, retired military rank, marital status, and parenthood status were recorded.

### Brief Form of the World Health Organization's Quality of Life Questionnaire (WHOQOL-BREF)

QOL of participants were evaluated using WHOQOL-BREF. The WHOQOL-BREF contains two items from the Overall Quality of Life and General Health facet and one item from each of the remaining 24 facets [14]. These facets are categorized into four domains: Physical Capacity (seven items), Psychological Well-Being (six items), Social Relationships (three items), and Environment (eight items). The WHOQOL-BREF (Taiwanese version) was developed in compliance with WHO guidelines [14-16]. This questionnaire includes 26 items translated from the English version and two items with local importance: "being respected" and "food availability", which are categorized into the Social Relationships and the Environment domains, respectively [14,17]. The WHOQOL-BREF Taiwanese version is a suitable QOL instrument for older people in Taiwan, with good validity and reliability [6]. All items were rated on a five-point scale, and the domain scores were calculated according to the formula provided in the user's manual, with a possible range of 4-20; the higher the score, the better the QOL [17]. In this study, the internal consistency of the WHOQOL-BREF domains were acceptable with a Cronbach's α of 0.74 in the physical and psychological domains, and 0.64 in social relationships, 0.61 in social relationships (TW) domains, and 0.72 in environment and environment (TW) domains. The internal consistency of the whole questionnaire was 0.90. The discriminative validity was also checked, and the results showed that scores in the Overall QOL, General Health, and four domains of the WHOQOL-BREF among institutionalized senior veterans who had chronic conditions or severe depressive symptoms were significantly lower than those who did not (*p *< 0.05).

### Subjective physical and mental health: The Short-Form 36 (SF-36)

Participants' subjective health status was evaluated using the SF-36 Taiwan version [18,19]. This 36-item questionnaire measures eight health concepts: Physical Functioning (10 items), Role Physical (four items), Bodily Pain (two items), General Health (five items), Vitality (four items), Social Functioning (two items), Role Emotional (three items), Mental Health (five items), and one item of Reported Health Transition [20]. The eight concepts could be grouped into Physical Component Summary (PCS) and Mental Component Summary (MCS) measures, which represent subjective physical health and mental health, respectively. PCS and MCS were rated according to the SF-36 users' manual [21].

### The social health questionnaire

The Inventory of Socially Supportive Behavior (ISSB) Taiwanese version was used to better evaluate residents' social health status. The amended ISSB Taiwanese version included 10 items evaluating the frequency of social support received from children, relatives, friends, social workers, and medical staff in the institute, as well as one item to evaluate residents' social support satisfaction [22]. In the questionnaire, 'social worker' was changed to 'social staff' to fit the characteristics of these VHs. The frequencies of social support items were categorized into never, occasionally, and often, and rated as 1, 2, and 3, respectively; satisfaction with social support was rated 1 if unsatisfied, 2 if did not matter, and 3 if satisfied. The score for each social support item and satisfaction ranged 10-30. Scores 10-16 were considered low, 17-23 as moderate, and >24 as a high indicator of social support or satisfaction [22].

### Objective health: chronic conditions, mental health, and functional status

Participants' objective health status was evaluated by recording their chronic conditions, symptoms of depression, and functional status of ADL and instrumental activities of daily living (IADL). Chronic conditions included hypertension, diabetes, heart disease, pulmonary disease, stroke, osteoarthritis, hyperlipidemia, renal disease, benign prostatic hypertrophy and cancer. Cognitive function were evaluated by Mini-Mental Status Examination (MMSE). The MMSE included 30 questions to evaluate orientation, registration, attention, calculation, recall, language and constructional ability with a score range 0-30, a lower score indicating the severity of cognitive impairment [12]. Depressive symptoms were evaluated using the short form of the Geriatric Depression Scale (GDS-15). The GDS-15 included 15 items with a score range 0-15; a score over 5 represents possible clinical depression, with a higher score indicating the severity of depression [23]. The ADL was evaluated using the Barthel index, with scores ranging 0-100 [4]. As meal and laundry services are provided in these institutions, the items used to evaluate participants' IADL function included six abilities (using the telephone, shopping, housekeeping, transportation, responsibility for own medications and ability to handle finances), with scores ranging 0-6; higher scores were indicative of better IADL ability [24].

### Procedures

The investigator conducted personal interviews with structured questionnaires in participants' rooms to collect related data. Due to the participants' age and the time-consuming nature of completing the questionnaires, if they felt tired or uncomfortable, the interview was paused until they indicated that they were ready to continue.

### Data analysis

Data analysis included descriptive and inferential statistics were performed using SPSS, version 17.0 (SPSS Inc., Chicago, IL, USA). Categorical variables are presented as frequencies and percentages, while continuous variables are presented as mean ± standard deviation. The internal consistency reliability of WHOQOL-BREF domains was estimated using Cronbach's coefficient. Discriminative validities for disease status (with or without chronic conditions) and severity of depressive symptoms (GDS-15 score <10 versus ≥ 10) were evaluated by Student's t-test. Univariate analyses were done, followed by socio-demographic characteristics including age, education level, retired military rank, marital status, parenthood status, and social health, ADL, IADL, MMSE and GDS-15, and number of chronic conditions were treated as the independent variables. Stepwise multiple linear regression analysis was used to assess the correlates of the participants' QOL in four domains.

## Results

### Subject characteristics and social health support

The average time to interview a participant was 40.38 ± 17.50 minutes (range 15-130 minutes); five subjects took a rest during the interview. Table 1 summarizes characteristics of participants and their social health support. The ages of the 260 residents ranged from 67 to 97 years, with an average of 82.91 ± 4.74 years. All subjects were male, 148 (56.9%) were retired as non-commissioned officers, 95 (36.5%) were literate, 125 (48.1%) had never married, and among who were married 88 (64.7%) had at least one child. The scores for their social support received from children, relatives, friends, social staff and medical staff produced a social support satisfaction score of 24.59 ± 2.80 (Table 1). Although these residents rated the social support provided by their children, relatives, friends, social and medical staff as low, they gave high satisfaction ratings to their social supports.

**Table 1:** Demographic characteristics of participants and their social health support.

	N	Mean ± SD or (%)
**Age**	260	82.91 ± 4.74
**Retired military rank**		
Enlisted man	48	(18.50)
Noncommissioned officer	148	(56.90)
Company officer	42	(16.20)
Field officer	11	(4.20)
Others	7	(2.70)
**Education level**		
Illiterate	49	(18.80)
Literate*	95	(36.50)
Elementary school	54	(20.80)
Junior high school	34	(13.10)
Senior high school	20	(7.70)
University	7	(2.70)
**Marital status**		
Never married	125	(48.10)
Married	49	(18.80)
Divorced	29	(11.20)
Widower	49	(18.80)
Others	8	(3.10)
**Children†**		
No	48	(35.3)
Yes	88	(64.7)
**Scores of social health support**		
Children's support	86	14.48 ± 3.81
Relatives' support	175	12.75 ± 3.34
Friends' support	260	14.45 ± 3.67
Social staff support	260	14.56 ± 2.42
Medical staff support	260	14.72 ± 2.62
Social satisfaction	260	24.59 ± 2.80

*Literate: literate but had never attended school

†The number did not sum up to 260 due to only 135 were ever married and 1 had an adopted child.

### Objective and subjective health

Table 2 summarizes information on chronic conditions, mental health, and functional status of the participants. The mean number of chronic conditions was 3.43 ± 1.91. The most common disease was osteoarthritis (64.4%), followed by hypertension (54%), benign prostatic hypertrophy (35.6%), and heart disease (33.6%). The mean scores of MMSE and GDS-15 were 25.82 ± 2.92 and 2.87 ± 2.64, respectively. There were 53 participants (20%) who scored over 5 points and needed further evaluation by psychiatric specialists. The mean ADL score was 97.73 ± 5.03, which indicated mild dependency, and the mean IADL score was 4.44 ± 1.46, which indicated that, except for preparing meals and laundry, these senior veterans still needed some help with IADL to live in the VH. The descriptive data for the SF-36 concepts scores are presented in Table 3. Physical functioning scored lowest among the eight concepts of the SF-36, and vitality scored the highest. When compared with the male norm aged ≥ 75 years in Taiwan [19], and ≥ 65 years in the United States [20], our participants scored low in all concepts of SF-36.

**Table 2:** Scores of objective health: Chronic conditions, mental health, and functional status measures.

Measure	N	%	mean ± SD
**Chronic conditions**	260		3.43 ± 1.91
Hypertension	135	54	
Diabetes	56	22.4	
Heart disease	84	33.6	
Pulmonary disease	39	15.6	
Stroke	15	6	
Osteoarthritis	161	64.4	
Hyperlipidemia	69	27.6	
Renal disease	24	9.6	
Benign prostatic hypertrophy	89	35.6	
Cancer	9	3.6	
Depressive symptoms*	16	6.4	
**Mental Health**			
MMSE	260		25.82 ± 2.92
GDS-15	260		2.87 ± 2.64
**Functional Status**			
ADL	260		97.73 ± 5.03
IADL	260		4.44 ± 1.46

*Depressive symptoms refer to clinically significant depressive symptoms: respondents identifying depression according to physician's previous diagnoses.

**Table 3:** Scores of subjective health (SF-36).

Concepts/Summary scores of SF-36	Senior veterans in Taiwan*	National Norms in Taiwan†	National Norms in USA‡
	N = 260	N = 308	N = 293
	Mean (SD)	Mean (SD)	Mean (SD)
Physical functioning (PF)	42.34 (11.71)	64.74 (25.13)	65.79 (28.31)
Role physical (RP)	48.25 (12.10)	51.54 (45.86)	59.72 (42.51)
Bodily pain (BP)	53.15 (10.09)	74.21 (23.78)	68.76 (25.73)
General health (GH)	43.11 (9.19)	54.19 (23.13)	58.62 (22.05)
Vitality (VT)	56.97 (9.03)	57.61 (19.83)	57.80 (22.55)
Social functioning (SF)	52.85 (8.61)	75.77 (22.65)	79.66 (26.00)
Role emotional (RE)	51.69 (9.73)	65.80 (43.30)	76.94 (37.48)
Mental health (MH)	52.46 (9.07)	72.98 (18.00)	77.37 (17.42)

*Senior veterans in Taiwan: results from present study

**†**National Norms in Taiwan: data drawn from [19]

**‡**National Norms in USA: data drawn from [20]

### QOL of institutionalized veterans assessed by WHOQOL-BREF

Descriptive results for the items and domains of the WHOQOL-BREF are shown in Table 4. Among the 28 items, only sexual activity (Q21) did not have a 100% response rate. Spirituality (Q6) scored the lowest of the 28 items, and positive feelings (Q5), dependence on medical substances, and medical aids (Q4) all scored below 3. The three top-scored items were pain and discomfort (Q3), food availability (Q28), and home environment (Q23). The mean domain scores of WHOQOL-BREF were 13.98 ± 2.16 for the physical domain, 13.10 ± 2.40 for the psychological domain, 13.53 ± 2.54 for the social relationships domain, 13.30 ± 2.28 for the social relationships domain of the Taiwanese version, 14.11 ± 1.70 for the environment domain, and 14.36 ± 1.71 for the environment domain of the Taiwanese version. The psychological domain was the lowest scoring domain, and environment was the highest domain. The addition of the national items of Taiwan, Q27 in the social relationships domain and Q28 in the environment domain, caused a slight decrease in the social relationships domain but a slight increase in the environment domain.

**Table 4:** Facet and domain scores of the WHOQOL-BREF (N = 260).

FaceFacets or Domains of WHOQOL-BREF	Mean	SD	Median	Mode
Q1 Overall Quality of Life	3.42	0.74	3	3
Q2 General health	3.35	0.82	3	3
**Physical domain**	**13.98**	**2.16**	**14.29**	**13.71**
Q3 Pain and discomfort*	4.31	0.89	5	5
Q4 Dependence on medical substances and medical aids*	2.72	1.37	2	2
Q10 Energy and fatigue	3.37	0.72	3	3
Q15 Mobility	3.18	0.90	3	3
Q16 Sleep and rest	3.42	0.96	4	4
Q17 Activities of daily living	3.73	0.66	4	4
Q18 Work capacity	3.74	0.66	4	4
**Psychological domain**	**13.10**	**2.40**	**13.33**	**12.67**
Q5 Positive feelings	2.71	1.10	3	3
Q6 Spirituality	2.67	1.10	3	3
Q7 Thinking, learning, memory, and concentration	3.08	0.80	3	3
Q11 Bodily image and appearance	3.21	0.65	3	3
Q19 Self-esteem	3.78	0.77	4	4
Q26 Negative feelings*	3.79	0.67	4	4
**Social relationships domain**	**13.53**	**2.54**	**13.33**	**12.00**
**Social relationships domain (TW)^‡^**	**13.30**	**2.28**	**13.00**	**12.00**
Q20 Personal relationships	3.42	0.78	3	3
Q21 Sexual activities	3.26	0.79	3	3
Q22 Social support	3.44	0.75	3	3
Q27 Being respected^**†**^	3.18	0.84	3	3
**Environment domain**	**14.11**	**1.70**	**14.00**	**14.00**
**Environment domain (TW) ^‡^**	**14.36**	**1.71**	**14.22**	**14.22**
Q8 Freedom, physical safety and security	3.67	0.68	4	4
Q9 Physical environment (pollution/noise/traffic/climate)	3.63	0.72	4	4
Q12 Financial resources	3.34	0.82	3	3
Q13 Opportunities for acquiring new information and skills	3.35	0.72	3	3
Q14 Participation in and opportunities for recreation/leisure activities	3.11	0.85	3	3
Q23 Home environment	3.82	0.66	4	4
Q24 Health and social care: accessibility and quality	3.79	0.67	4	4
Q25 Transport	3.52	0.82	4	4
Q28 Food availability^**†**^	4.10	1.04	4	5

* Q3, Q4, and Q26 are reverse-coded questions.

**†**The national item of WHOQOL-BREF Taiwan version Q27 'being respected' is included in the social relationships domain (TW), and Q28 'food availability' is included in the environment domain (TW).

**‡**Domains including national items of Taiwan.

### Correlates of the four QOL domains

The results of multiple linear regression models to analyze predictors of the four QOL domains are shown in Table 5. SF-36 was not included since its correlates were found with other independent variables in the model. Depressive symptoms and chronic conditions were inversely correlated with scores of physical domain QOL, while education level, retired military rank, and relatives' support were positively correlated, with standardized beta coefficients of -0.50, -0.33, 0.20, 0.20 and 0.18, respectively. This model explained 53% of the variance of physical domain QOL. Depressive symptoms and chronic conditions were inversely correlated to scores of psychological domain QOL, while retired military rank and relatives' supports were positively correlated, with standardized beta coefficients of -0.61, -0.24, 0.23 and 0.21, respectively. This model explained 49% of the variance of psychological domain QOL. Friends' support was a positive correlate of social relationships domain QOL, while depressive symptoms was inversely correlated, with standardized beta coefficients of 0.39 and -0.25, respectively. This model explained 23% of the variance of the social relationships domain QOL. Finally, friends' support and IADL were positive correlates of environment domain QOL, both with standardized beta coefficients of 0.29. This model explained 23% of the variance of environment domain QOL.

**Table 5:** Scores of multiple linear regression model for correlates of the four QOL domains.

Correlates	Un-standardized coefficients	Standardized coefficients	*T*	*P*	Co-linearity statistics	
	**B**	**Beta**			**Tolerance**	**VIF**

**Physical domain **(adjusted R^2 ^= 0.53)						
Constant	12.98		12.52	<0.0001		
GDS-15	-0.43	-0.50	-5.98	<0.0001	0.90	1.11
Chronic conditions	-0.45	-0.33	-3.77	<0.0001	0.82	1.22
Education level	0.68	0.20	2.12	<0.05	0.74	1.36
Retired military rank	0.62	0.20	2.33	<0.05	0.88	1.14
Relatives' support	0.12	0.18	2.14	<0.05	0.91	1.09
**Psychological domain **(adjusted R^2 ^= 0.49)						
Constant	12.81		12.50	<0.0001		
GDS-15	-0.58	-0.61	-7.01	<0.0001	0.91	1.10
Chronic condition	-0.36	-0.24	-2.81	<0.01	0.96	1.04
Retired military rank	0.79	0.23	2.65	<0.05	0.94	1.07
Relatives' support	0.16	0.21	2.53	< 0.05	1.00	1.00
**Social relationship domain (TW) **(adjusted R^2 ^= 0.23)						
Constant	10.13		8.11	<0.0001		
Friends' support	0.28	0.39	3.72	<0.0001	0.97	1.03
GDS-15	-0.24	-0.25	-2.43	<0.05	0.97	1.03
**Environment domain (TW) **(adjust R^2 ^= 0.23)						
Constant	7.58		4.33	<0.0001		
Friends' support	0.14	0.29	2.52	<0.05	0.79	1.27
IADL	0.28	0.29	2.49	<0.05	0.79	1,27

## Discussion

### Institutionalized senior veterans' subjective health and QOL

Senior veterans living in VHs are a special minority group in Taiwan. These seniors came from Mainland China as teenagers during military hostilities between the Kuomintang and the Chinese Communist Party after the end of World War II in 1945 [25]. Very few QOL instruments have been demonstrated to be suitable for use with the elderly living in institutions [7]; especially for institutionalized senior veterans [9] and for cross-cultural comparisons [7]. In this study, the mean QOL scores (Taiwanese version) of institutionalized senior veterans for the categories of physical, psychological and social relationships approached the 25th percentile, and the environment domain, the 75th percentile, compared to the male norms of Taiwan [17]. Compared to community-dwelling elderly living in the Shilin district of Taiwan, the senior veterans had significantly lower scores in four QOL domains [26]. Compared with results from the international field trial conducted by the WHOQOL group, the present participants had lower scores in the physical, psychological and social relationships domains [27]. VHs are likened to "total institution" LTC facilities, and they operate under different rules and models for daily living. Further evaluation may be needed to determine whether the "total institution" management model is one of the causes of this result. In the present research, environment domain scored high, which may be the result of the successful reconstruction of the living facilities and environment of VHs in recent years [28].

### Discrepancy between scores of social support and satisfaction

The results showed that the respondents rated the social support provided by their children, relatives, friends, social and medical staff as low, while giving high satisfaction ratings to their social supports. Most (81.2%) of our participants were never married, or were divorced or widowed. Among those who had been married, many had a foreign spouse from Mainland China. Among who had ever married, 64.7% had children, however, 60% of these children never supplied any support to their father due to residing outside of Taiwan. Factors associated with admission to LTC facilities included old age, living alone, low socio-economic status [29], marital status, financial means and children's opinions [30]. With reference to our study, senior veterans who are elderly, live alone and have a low socio-economic status tend to have little opportunity to live well in the community, and choose to be placed in VHs. According to their statements, lack of family ties may have contributed to their low social support ratings; however, this lack of support did not influence their current satisfaction within the VH. It is highly unlikely that respondents thought staff and families could be informed of their satisfaction ratings since the interviews were conducted anonymously and confidentiality was guaranteed. However, further in-depth personal interviews may be warranted to clarify this discrepancy.

### Correlates for QOL

Depressive symptoms (GDS-15) was a negative correlate for physical, psychologoical, and social relationships domains of QOL; in other words, for institutionalized senior veterans, the more depressive symptoms they had the lower their QOL scores. Several studies have shown that major depression or subsyndromal symptoms of depression are an important predictor for impaired QOL [31-34]. Furthermore, depression is also associated with a worsened outcome of functional decline and mortality in the elderly [35]. Therefore, a full-scale screening, comprehensive evaluation and treatment of depression for residents in VHs are necessary to improve their QOL.

The results of the present study show that relatives' support is a positive correlate of the physical and psychological domains of QOL. Support of friends was also positively correlated to social relationship, and environmental domains of QOL. Social support is important to health outcomes and has a positive effect on QOL [28,29], and good social relationships are the most commonly reported constituent influencing QOL in the elderly [30]. In the present study, however, these senior veterans received limited social support from their relatives and friends. As these seniors originally came from different provinces of China in their teenage years, many of them never married, never fostered a child, and had limit friendships. Determining how to help these senior veterans to effectively improve their social support, especially support from relatives and friends, is critical to their QOL.

Number of chronic conditions was a negative predictor for physical, psychological, and environmental domain scores of QOL, and similar findings have been reported for elderly persons living in the community [36], living alone [37] or in institutions [7,8,38]. The present participants had an average of three chronic diseases. A systematic review reported an inverse relationship between multicomorbidity and physical domain QOL in primary care [39], and some studies have revealed a similar relationship in patients with four or more conditions for the psychological domain [39]. This study, showed similar results for elderly living in VHs, with chronic disease control and prevention of multiple comorbidities being important factors of QOL. Retired military rank was positively correlated to physical and psychological domain scores; the higher the retired rank, the better the QOL scores. It seems that, although these veterans were retired, their military rank as a sign of socioeconomic status still played some role in daily living in the VHs.

When analyzing the adjusted value for the square of coefficient of determination (R^2^) in the regression models for the four QOL domain scores, the R^2 ^for the physical domain had the highest value (0.53), with low values for social relationship (0.23) and environmental (0.25) domains. It seems that there are still other factors that may influence these institutionalized senior veterans' QOL, especially in the social relationship and environmental domains.

### Limitations

This study had several limitations. First, the cross-sectional study design could only clarify associations, and not causal relationships, between correlates and QOL. Second, due to the quantitative design of our study, these issues could not be explored through in-depth interviews. Therefore, a qualitative study should be conducted in the future to more deeply explore other issues that could influence veterans' perspectives on health conditions and QOL, such as meaningful life events and life satisfaction. Third, the social and environmental domains of WHOQOL-BREF were not well-explained by the present model, so there may be other important factors we did not extract. Therefore, further in-depth personal interviews are warranted to clarify WHOQOL-BREF. Fourth, since the present participants were all male veterans, it is uncertain whether these results can be applied to female veterans. Thus, further study should be conducted to evaluate the health status and QOL of the female veterans in Taiwan. Fifth, the questionnaire instruments were only administered to senior veterans who received personal care in VHs. There was little non-response bias, as demonstrated by the lack of significant difference in basic socio-demographic characteristics between participants and non-respondents. However, those residing in nursing care units (Barthel Index lower than 90 or needing skilled nursing care) were not included.

## Conclusions

We conclude that senior male veterans living in veterans' homes in southern Taiwan are in a state of low subjective health. They also have lower QOL levels in the physical, psychological and social relationship domains as compared with male norms and community-dwelling elderly in Taiwan. Symptoms of depression are a salient factor inversely affecting senior veterans' QOL in the physical, social relationships and environmental domains. Receiving social support from relatives and friends is positively correlated to the physical and psychological, social relationships and environmental domains, respectively, and the number of chronic conditions has an inverse relationship on QOL scores in the physical, and psychological domains. Helping senior veterans to effectively treat and ease their depressive symptoms, improving their social support, and controlling chronic disease appear to be the critical factors in improving their QOL. In-depth personal interviews are needed to clarifying the discrepancy between their ratings of social supports and support satisfaction. We also need to determine whether the so-called "total institution" management model of veterans' homes is one cause of veterans' low QOL.

## Abbreviations

ADL: activities of daily living; GDS-15: short form of the Geriatric Depression Scale; IADL: instrumental activities of daily living; ISSB: Inventory of Socially Supportive Behavior; LTC: long-term care facilities; MCS: Mental Component Summary; PCS: Physical Component Summary; QOL: quality of life; SF-36: The Short-Form 36; VH: veterans' home; WHOQOL-BREF: Brief Form of the World Health Organization's Quality of Life Questionnaire.

## Competing interests

The authors declare that they have no competing interests.

## Authors' contributions

HTC conceived and carried out the study, performed the statistical analysis, interpreted findings and drafted the manuscript. LFL is the principal investigator in the design of the study, interpreted findings and drafted the manuscript. CKC participated in data collection and statistical analysis. SJH, LKC and FHL helped to interpret findings. All authors have read and approved the final manuscript.
